# Body temperature as a predictor of mortality in COVID-19

**DOI:** 10.1038/s41598-023-40414-z

**Published:** 2023-08-16

**Authors:** Shuhei Uchiyama, Tomoki Sakata, Serena Tharakan, Kiyotake Ishikawa

**Affiliations:** 1 Cardiovascular Research Institute, Icahn School of Medicine at Mount Sinai, 1 Gustave L. Levy Place, Box 1014, New York, NY USA; 2 Department of Medicine, Mount Sinai Beth Israel, New York, NY USA

**Keywords:** Fever, Epidemiology

## Abstract

It remains uncertain if body temperature (BT) is a useful prognostic indicator in coronavirus disease 2019 (COVID-19). We investigated the relationship between BT and mortality in COVID-19 patients. We used a de-identified database that prospectively collected information from patients screened for COVID-19 at the Mount Sinai facilities from February 28, 2020 to July 28, 2021. All patients diagnosed with COVID-19 that had BT data were included. BT at initial presentation, maximum BT during hospitalization, comorbidity, and vaccination status data were extracted. Mortality rate was assessed as a primary outcome. Among 24,293 cases, patients with initial BT below 36 °C had higher mortality than those with BT of 36–37 °C (*p* < 0.001, odds ratio 2.82). Initial BT > 38 °C was associated with high mortality with an incremental trend at higher BT. In 10,503 in-patient cases, a positive association was observed between mortality and maximum BT except in patients with BT < 36 °C. Multiple logistic regression analyses including the comorbidities revealed that maximum BT was an independent predictor of mortality. While vaccination did not change the distribution of maximum BT, mortality was decreased in vaccinated patients. Our retrospective cohort study suggests that high maximum BT is an independent predictor of higher mortality in COVID-19 patients.

## Introduction

Severe acute respiratory syndrome coronavirus 2 (SARS-CoV-2) infection, known as coronavirus disease 2019 (COVID-19), continues to be the most pressing public health concern worldwide. National guidelines for COVID-19 care were published in April 2020^1^ and have been updated multiple times to reflect evolving evidence. Some studies have identified risk factors for severe or fatal cases allowing for prognostication and implementation of prophylactic measures^2–5^.

We previously reported that high maximum body temperature (BT) during COVID-19 infection was associated with poor prognosis^6^. However, the study was published in June 2020 and was limited in scope, because the original cohort was based on fewer patients and did not account for possible confounders. In addition, vaccination against COVID-19 was initiated in the United States in December 2020 after our first publication. Thus, we could not study how vaccination status might impact the relationship between BT and mortality.

Despite high incidence of fever in COVID-19, its impact on mortality remains unclear. Based on our preliminary study, we hypothesized that both high and low body temperatures are predictors of mortality in patients with COVID-19. Accordingly, we investigated the relationship between BT and mortality in COVID-19 patients using a larger database than in our previous study and also included a new analysis of a vaccinated population.

## Methods

### Study population

This was a retrospective cohort study conducted in accordance with the Strengthening the Reporting of Observational Studies in Epidemiology (STROBE) guidelines^7^. The IRB at the Icahn School of Medicine at Mount Sinai (Program for the Protection of Human Subjects, Institutional Review Boards, Mount Sinai Health System) reviewed the study and data collection protocol (IRB-20-03579) and deemed scientific publication of the de-identified patient information exempt from the IRB review and informed consent from individual patients. The study was approved by the Research Ethics Committee at the Icahn School of Medicine at Mount Sinai, and was conducted in accordance with the principles of the Declaration of Helsinki. At the Icahn School of Medicine at Mount Sinai, a de-identified COVID-19 patient database was generated and updated every week, and made available to the Mount Sinai Health System research community. Patients who were screened for or diagnosed with COVID-19 at the Mount Sinai facilities in New York were entered in the database. We extracted data from patients with confirmed SARS-CoV-2 infection by positive reverse transcription polymerase chain reaction from February 28th, 2020, to July 28th, 2021.

### Data collection

Demographics, disease diagnoses, comorbidities, temperature data and outcome data were extracted from Mount Sinai Data Warehouse COVID-19 Electronic Health Record, which is available for clinicians in the Mount Sinai Health System. Patients were considered alive if they were discharged from the hospital alive or remained hospitalized at the time of the analysis (July 28th, 2021). Analyzed comorbidities included asthma, chronic obstructive pulmonary disease, hypertension, obstructive sleep apnea, diabetes mellitus, chronic kidney disease, human immunodeficiency virus infection, cancer, coronary artery disease, atrial fibrillation, heart failure, chronic viral hepatitis, alcoholic/non-alcoholic liver disease, Crohn's disease, ulcerative colitis and peripheral vascular disease. Mortality rate was assessed as a primary outcome. Outpatient deaths were recorded if patients presented to our facilities and expired before admission, or their death was reported by outside hospitals or family. For vaccination status, we grouped patients based on the number of doses, and did not distinguish between the three types of vaccines available in the U.S. (Pfizer, Moderna, and Johnson and Johnson).

### Statistical analysis

Continuous variables are presented as mean ± standard deviation or median with interquartile range. Categorical variables are expressed as absolute number of patients with percentage. When chi-square test was conducted between multiple groups, Holm's method was used to adjust for multiple comparisons. Kaplan–Meier estimators with Log-rank tests were performed to estimate survival. To determine contributing factors for elevated maximum BT, a linear regression analysis with the ordinary least square method was performed. In linear regression, normality of the residuals and homoskedasticity were verified with histograms, Q–Q plots and scatter plots of residuals and fitted values. The values which are clinically unreasonable were regarded as outliers and removed from analysis. To determine the impact of maximum BT and other comorbidities on mortality, logistic regression analyses were performed. For multivariate logistic regression, every factor with *P* < 0.05 in the linear regression analysis was included. Multicollinearity between continuous variables was determined using correlation coefficients, and either of the variables was excluded from the independent variables of multivariate analyses if it was greater than 0.8. All statistical tests were 2-tailed. Statistical analyses were performed using JMP version 12.0 (SAS Institute Inc, Cary, NC).

### Ethics approval and consent to participate

The IRB at the Icahn School of Medicine at Mount Sinai reviewed the data collection protocol and deemed scientific publication of the de-identified patient information exempt from the IRB review.

## Results

**Figure 1:**
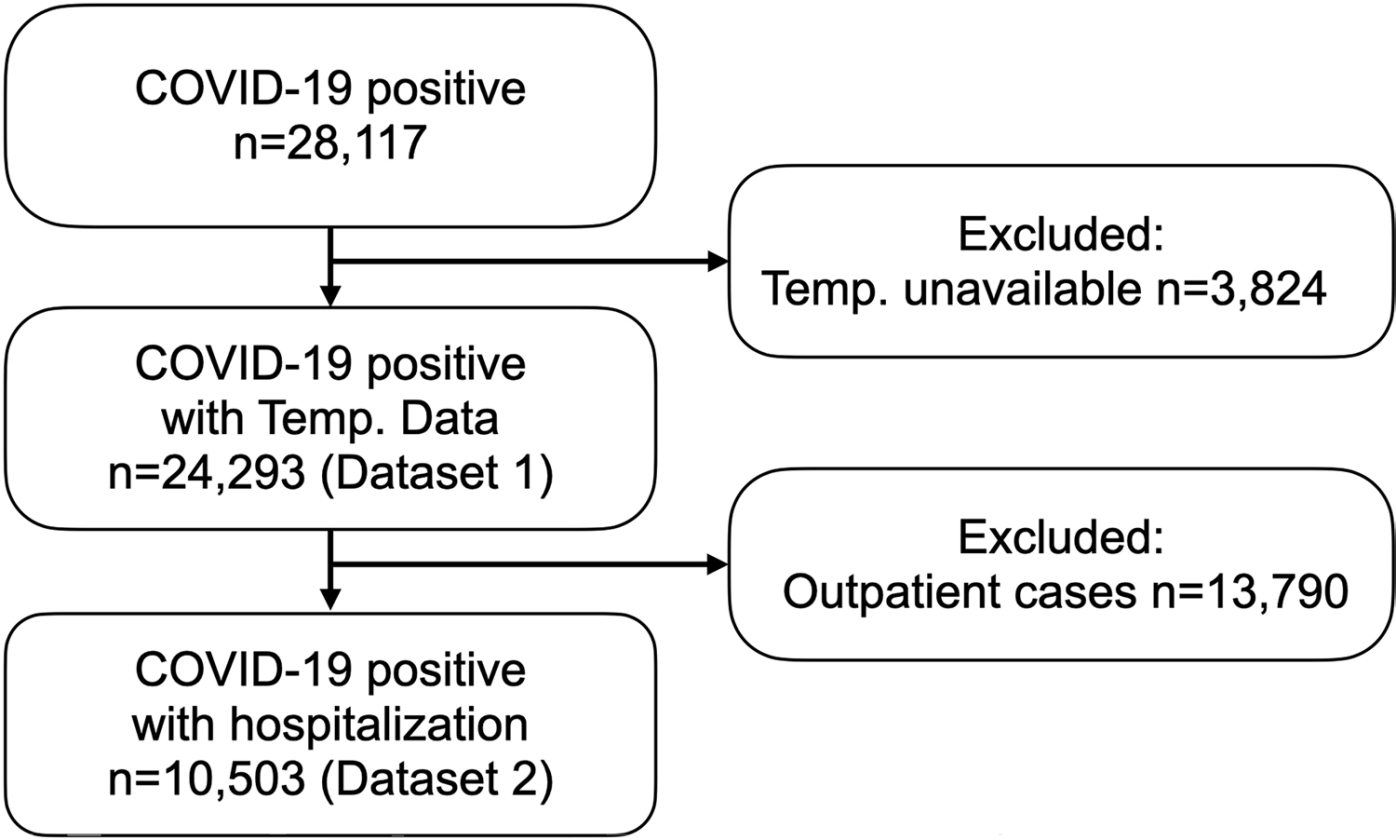
Flow chart of included patient numbers. Dataset 1 includes all SARS-CoV-2 positive patients with body temperature data. Dataset 2 includes hospitalized patients with follow-up body temperature data.

Figure 1 shows the sample numbers in a flow chart. The database consisted of 28,117 cases. Among them, 3824 patients were excluded due to missing BT data, leaving 24,293 patients (Dataset 1). To study the impact of maximum BT during the course of COVID-19, outpatients were excluded and 10,503 in-patient cases were included in Dataset 2. To assess the relationship between initial BT at the first encounter and mortality, both in-patients and outpatients were included (Dataset 1). However, outpatients were excluded for assessing the relationship between maximum BT during the course of hospitalization and mortality (Dataset 2). The dataset showed that other than 6053 patients who were discharged home and 2447 patients who expired, there were 2003 patients who were transferred to other institutions or to other departments and facilities within Mount Sinai Health System. Patient characteristics for both Datasets are shown in Table 1.

**Table 1:** Patient characteristics in Dataset 1 and Dataset 2.

	Dataset 1	Dataset 2
Patients number	24,293	10,503
Age (SD) y.o	52.48 (21.50)	64.30 (17.68)
Sex (male, %)	12,234 (50.4%)	5679 (54.1%)
Initial temperature (Celsius, SD)	36.96 (0.68)	37.15 (0.92)
Maximum temperature (Celsius, SD)		38.21 (0.96)
Asthma	1.87 (5.7%)	623 (5.9%)
Chronic obstructive pulmonary disease	595 (2.4%)	474 (4.5%)
Hypertension	5596 (23.0%)	3815 (36.3%)
Obstructive sleep apnea	443 (1.8%)	250 (2.4%)
Diabetes mellitus	3438 (14.2%)	2473 (23.5%)
Chronic kidney disease	1583 (6.5%)	1225 (11.7%)
Human immunodeficiency virus infection	354 (1.5%)	177 (1.7%)
Cancer	1569 (6.5%)	941 (9.0%)
Coronary artery disease	2001 (8.2%)	1512 (14.4%)
Atrial fibrillation	1095 (4.5%)	885 (8.4%)
Heart failure	1189 (4.9%)	956 (9.1%)
Viral hepatitis	174 (0.7%)	112 (1.1%)
Alcoholic or nonalcoholic liver disease	475 (2.0%)	307 (2.9%)
Crohn's disease	78 (0.3%)	39 (0.4%)
Ulcerative colitis	77 (0.3%)	48 (0.5%)
Peripheral vascular disease	663 (2.7%)	491 (4.7%)
Body mass index, (kg/m^2^, SD)	28.50 (7.37)	28.65 (7.54)
Vaccination		
0		9470 (90.2%)
1		208 (2.0%)
2		819 (7.8%)

**Figure 2:**
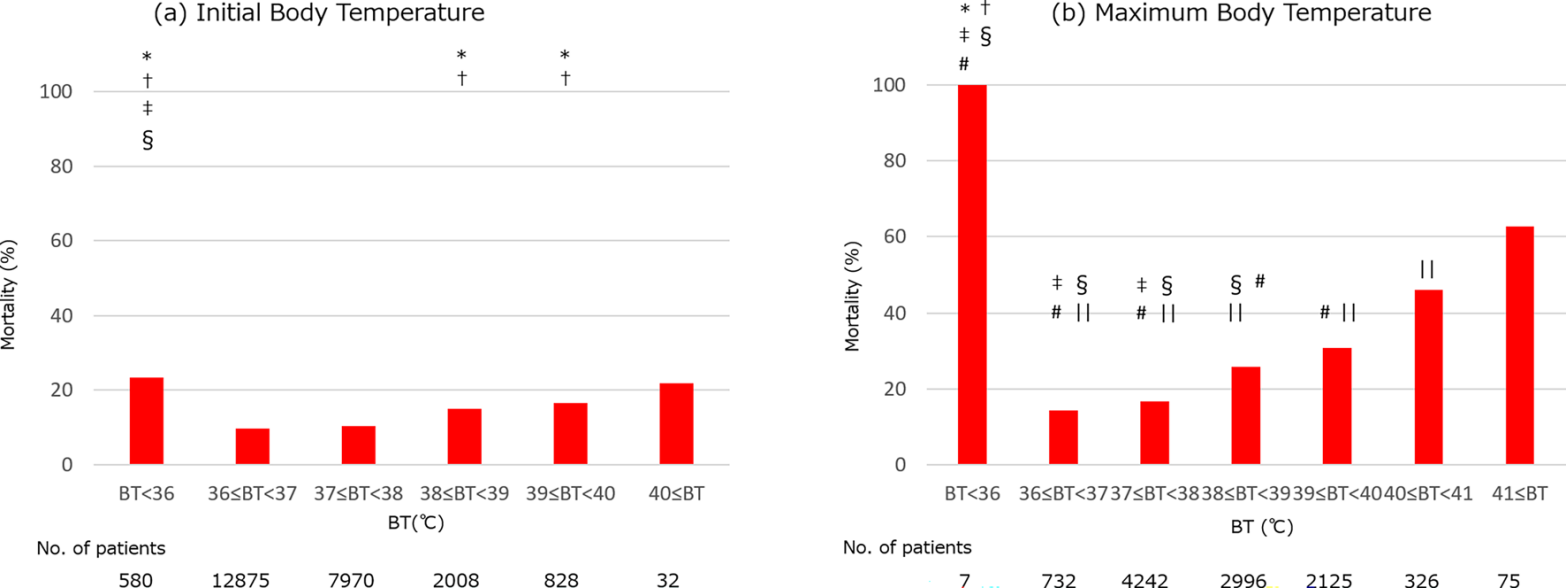
Mortality based on initial BT (**a**) and maximum BT (**b**). Relationship between initial BT and mortality was studied in all population with BT data (n = 24,293) (**a**), whereas relationship between maximum BT and mortality was studied in in-patients only (n = 10,503) (**b**). *p < 0.05 against 36–37 °C group, † *p* < 0.05 against 37–38 °C group, ‡ *p* < 0.05 against 38–39 °C group, § *p* < 0.05 against 39–40 °C group, # *p* < 0.05 against 40–41 °C group, and || *p* < 0.05 against > 41 °C group on Chi-square with Holm’s test (see supplemental materials). BT = body temperature.

Figure 2 shows the mortality for every 1 °C BT difference at the initial encounter (a) and maximum BT observed during admission (b). Since there was only one patient with initial BT of 41 °C or greater, this patient was added to the group of initial BT of 40–41 °C. Patients with low initial BT (< 36 °C) had a higher mortality compared to those within 36–37 °C (*p* < 0.0001, OR 2.82, 95% confidence interval (CI) 2.31–3.45). There was also an incremental mortality trend in higher initial BT patients. Meanwhile, mortality showed a more pronounced association with maximum BT compared to initial BT. Mortality increased for every 1 °C increase in maximum BT above 38 °C (Fig. 2b) with OR 1.28 (95%CI 1.13–1.45) for maximum BT of 38–39 °C versus 39–40 °C, OR 1.92 (95%CI 1.51–2.43) for 39–40 °C versus 40–41 °C, and OR 1.97 (95%CI 1.18–3.30) for 40–41 °C vs above 41 °C. Mortality was greater than 60% for those above 41 °C. Furthermore, all seven patients who were unable to raise BT above 36 °C died.

**Figure 3:**
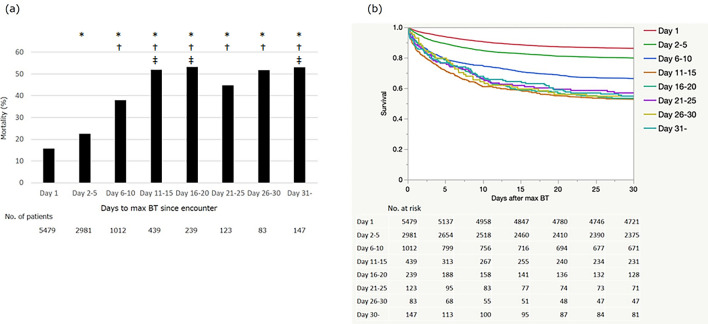
Mortality rate in relation to days until maximum BT. Patients were categorized based on the number of days until reaching maximum BT after initial encounter (**a**). Survival curves were drawn from the time of maximum BT with an assumption that discharged patients survived for 30 days (**b**). The *p* value was < 0.001 on Log-rank test. * *p* < 0.05 against day 1 group, † *p* < 0.05 against day 2–5 group, ‡ *p* < 0.05 against day 6–10 group on Chi-square with Holm’s test. BT = body temperature.

Next, we investigated the relationship between the time to reach maximum BT and mortality. Figure 3 shows that BT increase after admission was associated with worse prognosis compared to those presenting with maximum BT on day 1. Mortality increased in patients with longer time to maximum BT, plateauing after 10 days. We then examined if certain comorbidities were associated with maximum BT. As shown in Supplemental Table 1, single linear regression analysis demonstrated that male sex, body mass index, history of hypertension, obstructive sleep apnea, diabetes mellitus, chronic kidney disease, malignancy, coronary artery disease, atrial fibrillation, and liver disease were positively correlated with maximum BT, whereas age was negatively correlated with maximum BT. To examine factors that influenced mortality, we conducted a logistic regression analysis with in-hospital death as an objective variable. As shown in Table 2, based on the odds ratio, confidence interval, and clinical significance, we determined that age, male sex, history of chronic obstructive pulmonary disease, hypertension, diabetes, chronic kidney disease, cancer, coronary artery disease, atrial fibrillation, heart failure, and peripheral vascular disease were associated with increased mortality. It also demonstrated that asthma, Crohn's disease, ulcerative colitis, and vaccination against COVID-19 were associated with lower mortality. We next conducted a multiple logistic regression analysis using the factors with *p* < 0.05 in the univariate analysis. Maximum BT remained an independent prognostic indicator of mortality even after adjusting for these factors, with an odds ratio of 1.88 (*p *< 0.0001, 95%CI 1.78–1.99) for every one degree increase of maximum BT (Table 2). Multiple logistic regression analysis using the parameters that showed high correlation to maximum BT also showed similar results (Supplemental Table 2).

**Table 2:** Logistic regression analyses for in-patient death.

	Univariate analysis	*p* value	Multivariate analysis	*p* value
	Odds ratio (95% CI)		Odds ratio (95% CI)	
Maximum BT	1.54 (1.47–1.62)	< 0.0001	1.88 (1.78–1.99)	< 0.0001
Age	1.05 (1.05–1.06)	< 0.0001	1.06 (1.06–1.07)	< 0.0001
Sex (male)	1.13 (1.03–1.24)	0.0096	1.19 (1.08–1.32)	0.0009
Asthma	0.65 (0.52–0.81)	< 0.0001	0.82 (0.64–0.95)	0.12
Chronic obstructive pulmonary disease	1.69 (1.39–2.05)	< 0.0001	1.32 (1.05–1.65)	0.017
Hypertension	1.36 (1.24–1.49)	< 0.0001	0.80 (0.71–0.91)	0.0005
Obstructive sleep apnea	0.97 (0.71–1.30)	0.85	–	–
Diabetes	1.23 (1.11–1.37)	< 0.0001	1.14 (1.01–1.30)	0.041
Chronic kidney disease	1.77 (1.56–2.02)	< 0.0001	1.63 (1.39–1.91)	< 0.0001
Human immunodeficiency virus infection	0.72 (0.48–1.05)	0.099	–	–
Cancer	1.39 (1.20–1.61)	< 0.0001	1.25 (1.05–1.48)	0.011
Coronary artery disease	1.77 (1.57–1.99)	< 0.0001	1.28 (1.10–1.48)	0.0010
Atrial fibrillation	1.91(1.65–2.21)	< 0.0001	1.26 (1.06–1.49)	0.0094
Heart failure	1.64 (1.42–1.89)	< 0.0001	1.08 (0.90–1.29)	0.40
Viral hepatitis	0.76 (0.46–1.20)	0.25	–	–
Alcoholic or nonalcoholic liver disease	1.19 (0.91–1.53)	0.19	–	–
Crohn’s disease	0.09 (0.005–0.40)	0.016	0.12 (0.007–0.65)	0.0085
Ulcerative colitis	0.30 (0.09–0.74)	0.021	0.43 (0.12–1.14)	0.094
Peripheral vascular disease	1.29 (1.05–1.58)	0.014	0.80 (0.63–1.00)	0.054
Body mass index	0.66 (0.41–1.04)	0.074	–	–
Vaccination 0 → 1	0.29 (0.18–0.46)	< 0.0001	0.21 (0.13–0.34)	< 0.0001
Vaccination 1 → 2	0.15 (0.07–0.30)	< 0.0001	0.15 (0.07–0.32)	< 0.0001

Simple logistic regression (a) and multivariate logistic regression analysis (b) using the significant parameters in (a).

*BT* body temperature, *CI* confidence interval.

**Figure 4:**
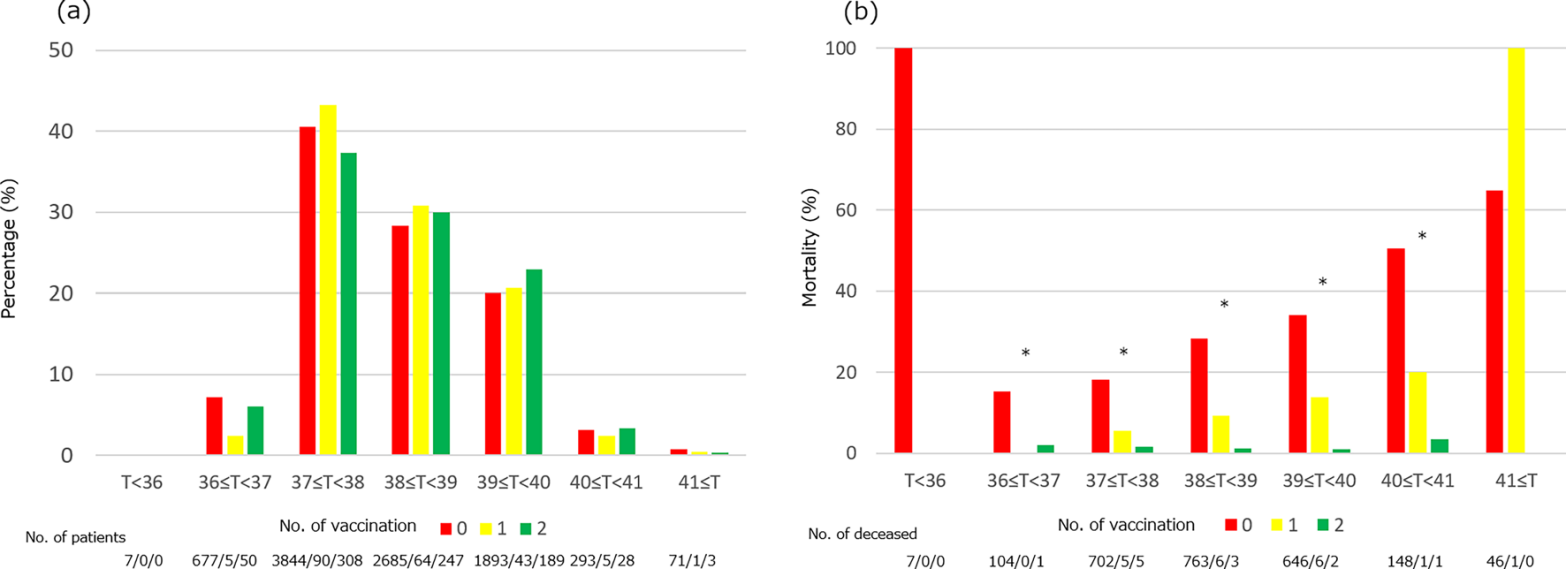
Maximum BT and mortality with and without vaccination. (**a**) Distribution of maximum BT and (**b**) mortality in patients grouped by vaccination and maximum BT. The distribution of maximum BT for each vaccination status did not show significant differences. In contrast, mortality was reduced for each maximum BT category when the patients were vaccinated. Chi-square test for the entire group in (**a**) returned a *p* value of 0.79. In (**b**), * demonstrates *p* < 0.05 among the three vaccination groups. The *p* value in maximum BT ≥ 41 °C was 0.056. BT = body temperature.

Finally, we investigated the relationship between vaccination against SARS-CoV-2, maximum BT, and death in in-patients (Fig. 4). In dataset 2, there were 1027 patients with more than one vaccination at the time of first encounter. We performed Chi-square test to compare the population ratio of each BT group in patients with no, one, and two vaccinations. There was no difference in maximum BT distribution relative to non-vaccinated population regardless of the number of vaccinations (Fig. 4a, *p* = 0.79). Next, we performed Chi-square tests in each temperature group to assess the gap in mortality rate among patients with no vaccination, one vaccination, and two vaccinations.

Mortality was highest in patients with no history of vaccination in all temperature groups, except for the group above 41 °C in which there was only one vaccinated patient.

As we were not able to track the final outcomes of those who were transferred to other institutes, facilities or departments, we conducted sensitivity analysis creating an additional dataset which excluded those patients from dataset 2 (dataset 3, patient characteristics in Supplemental Table 3). The same analyses as dataset 2 were performed and revealed that high maximum body temperature is still associated with increased mortality and vaccination reduced mortality regardless of maximum body temperature (Supplemental Figs. 1–3, Supplemental Tables 4–6). The details of Holm’s correction for all analyses are shown in Supplemental Tables 7–18.

## Discussion

In this study, our data suggested that BT at the time of hospital presentation as well as maximum BT during the course of disease were associated with mortality in COVID-19 patients, as described in Fig. 2. Specifically, both high and low initial BT were associated with poor prognosis and the trend was even clearer with maximum BT in hospitalized patients. We also found that reaching maximum BT in the later phase of hospitalization was associated with higher mortality. Maximum BT remained an independent predictor of in-hospital death after adjusting for patient characteristics and comorbidities. In addition, while vaccination status did not change the distribution of maximum BT after admission, we found that there was a reduced mortality in the vaccinated population as shown in Fig. 4. Mortality for any given maximum BT was lower across all temperature range with a trend showing lower mortality for patients with two vaccinations.

Consistent with our previous findings^6^, we demonstrated that low BT (< 36 °C) at the time of hospital presentation is associated with high mortality rate. Although the number of patients who remained hypothermic after hospitalization was limited, all seven patients died. These results indicate that hypothermia is a sign of poor prognosis in COVID-19 patients, especially when it is sustained. Indeed, hypothermia is a known predictor of poor prognosis in severely ill patients admitted to the intensive care unit^8,9^. Recently, Maait et al.^10^ reported that COVID-19 patients with hypothermia at the time of intensive care unit administration had more than 2-fold higher mortality. Our mortality data are similar to theirs and confirmed that hypothermia is a sign of worse prognosis not only for patients in an intensive care unit, but also for all hospitalized patients using a much larger dataset.

Elevated BT was also associated with increased mortality for both initial BT and maximum BT compared to normothermia patients. This trend was not clear in the previous analysis in June 2020 for initial BT, probably because of the smaller sample size at that time^6^. In contrast, both our previous and current studies indicate that the higher the maximum BT, the higher the mortality in hospitalized COVID-19 patients. Using this larger sample size, we demonstrated here that more than half of patients with maximum BT > 41 °C died. After adjusting for patient demographics and comorbidities, maximum BT remained an independent predictor of mortality together with several known risk factors such as older age, chronic kidney disease, heart diseases and cancer^11–13^.

Secondly, our data suggested that prognosis is worse if the maximum BT is reached in the later phase of COVID-19 admission, especially after 10 days. This likely reflects sudden worsening in some uncontrolled COVID-19 patients, and BT elevation might be one of the signs associated with late onset severe disease. It is also possible that late onset fever is related to the complications of COVID-19. For example, it has been reported that patients with severe COVID-19 infection requiring mechanical ventilation are more likely to develop ventilator-associated pneumonia than patients with other etiologies requiring ventilation^14^. Other complications include deep vein thrombosis^15^ and sepsis due to secondary infection. We also examined the factors that were related to maximum BT. Younger age, male sex, and higher body mass index were associated with higher maximum BT along with some comorbidities such as hypertension and diabetes (Supplement Table 1). After adjusting for these factors, we still found that maximum BT was an independent predictor of mortality in COVID-19 patients. It is noteworthy that every one degree increase in maximum BT was associated with 1.88-fold higher mortality (Table 2), suggesting its high clinical significance as a predictor of mortality. Interestingly, age was inversely correlated with maximum BT, while it was positively correlated with mortality. This is consistent with a previous study, which showed that age is inversely correlated with mean BT in patients hospitalized for pneumonia^16^.

Third, while this study showed that vaccination reduced mortality in hospitalized COVID-19 patients, it also suggested that the vaccine might not have a significant effect on maximum BT. Linear regression analysis on maximum BT also failed to show a clear relationship between vaccination and maximum BT in our population, further supporting a lack of interaction. Although lower risk of hospitalization and decreased mortality have been reported in the vaccinated population^17^, our data are unique in investigating the relationship between vaccination status and fever. Our data indicates that high fever could develop in vaccinated COVID-19 patients with more favorable outcome, suggesting a beneficial effect of vaccination on relationship between maximum BT and mortality.

Our study has limitations. First, our de-identified database did not include methods of temperature measurement and antipyretic information. Because our study only describes association and does not necessarily indicate a causal relationship, whether active BT management is beneficial for COVID-19 patients remains unclear. Recently, the interleukin-6 receptor inhibitor tocilizumab demonstrated improved clinical outcome in severe COVID-19 patients^18^. Interleukin-6 is a well-established fever-generating molecule in inflammation and is known to be upregulated in COVID-19 patients^19^. Unfortunately, whether tocilizumab affected fever was not studied. Nevertheless, these results, together with ours, underscore the need for future investigation on BT management in COVID-19. Second, our study included all patients from early 2020, when there were no guidelines for COVID-19 care. The treatment at the time may be quite different from current treatments, which may have affected the results. Third, since the database included clinical information from all Mount Sinai facilities, availability of medical devices in each facility could have affected the outcome. For example, some of the hospitals have extracorporeal membrane oxygenation, which could be indicated for patients with severe ARDS secondary to COVID-19 infection. Fourth, there was some difference in the prevalence of comorbidities between outpatient population and in-patient population as shown in Table 1, which precludes us from making a simple comparison of these two settings.

Fifth, we only categorized patients according to the number of vaccination doses, and did not group them based on the three different vaccines available in the U.S. Thus our data might be affected by the effectiveness of different vaccines, particularly with single-dose Ad26.COV2.S vaccine. In addition to these limitations, our analysis is subjected to several potential biases. For example, there were a significant number of patients (3824) with missing BT data, although demographic data of these patients was similar to those of the patients included in the analysis, suggesting the missing data occurred at random. In addition, we assumed that all patients hospitalized at the time of data analysis were alive. Although the number of admitted patients is expected to be small relative to the whole population, some of those patients might have died after this analysis. Additionally, mortality of outpatients in Dataset 1 could have been underestimated by missed reporting.

Finally, it is possible that the result of each analysis bears undetected confounding factors that can affect the relationship between each comorbidity and mortality^20^.

Despite the limitations noted above, we believe that our findings on the relationship between body temperature and mortality is clinically valuable for predicting the outcome and to guide the care of patients with COVID-19.

## Conclusion

Our retrospective cohort study revealed that high maximum BT is an independent prognostic factor for worse prognosis in COVID-19 infection. Our study also suggested that although vaccination lowers the mortality, it does not affect maximum BT.

### Supplementary Information

Supplementary Information.

## Data Availability

The datasets generated and/or analyzed during the current study are not publicly available due to institutional policy but are available from the corresponding author on reasonable request and with permission of the Icahn School of Medicine at Mount Sinai.

## Abbreviations

BT: Body temperature
COVID-19: Coronavirus disease 2019
SARS-CoV-2: Severe acute respiratory syndrome coronavirus 2
