# Supplementary material for: Body temperature as a predictor of mortality in COVID-19
Source: Sci Rep. 2023 Aug 16;13:13354. doi: 10.1038/s41598-023-40414-z (PMC10432378; doi:10.1038/s41598-023-40414-z)
Supplement: Supplementary file 1 — Supplementary Information. [file 41598_2023_40414_MOESM1_ESM.docx]

**Supplemental Figure 1: Relationship between maximum BT and mortality in inpatients who were discharged home or expired**


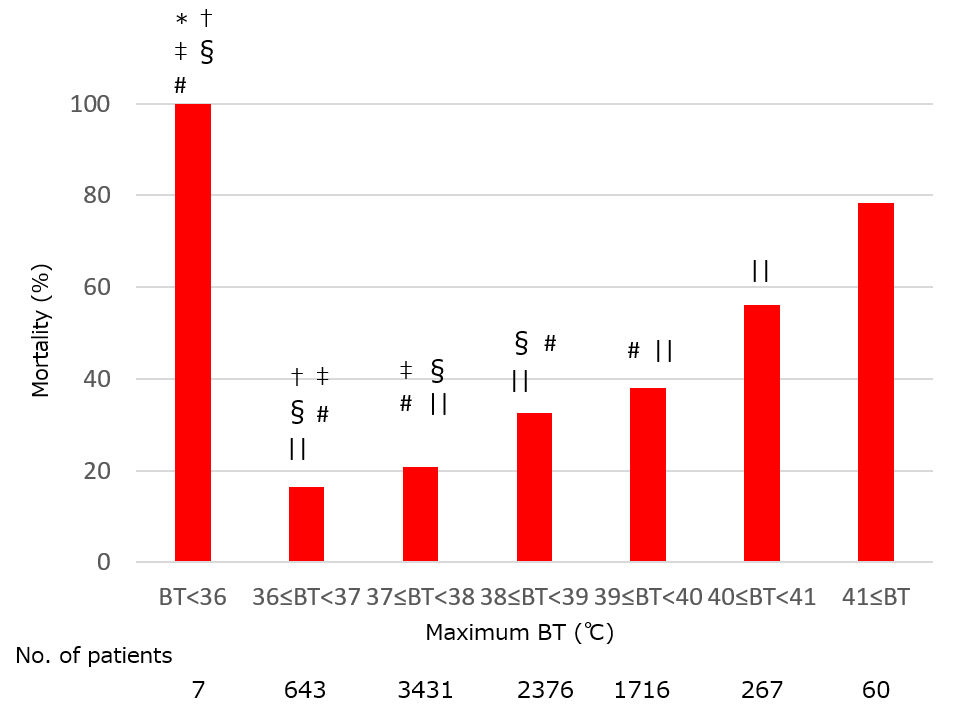


* p<0.05 against 36-37°C group, † p<0.05 against 37-38°C group, ‡ p<0.05 against 38-39°C group, § p<0.05 against 39-40°C group, # p<0.05 against 40-41°C group, and || p<0.05 against >41°C group on Chi-square with Holm’s test. BT = body temperature

**Supplemental Figure 2: Mortality rate in relation to days until maximum BT in inpatients who were discharged home or expired**


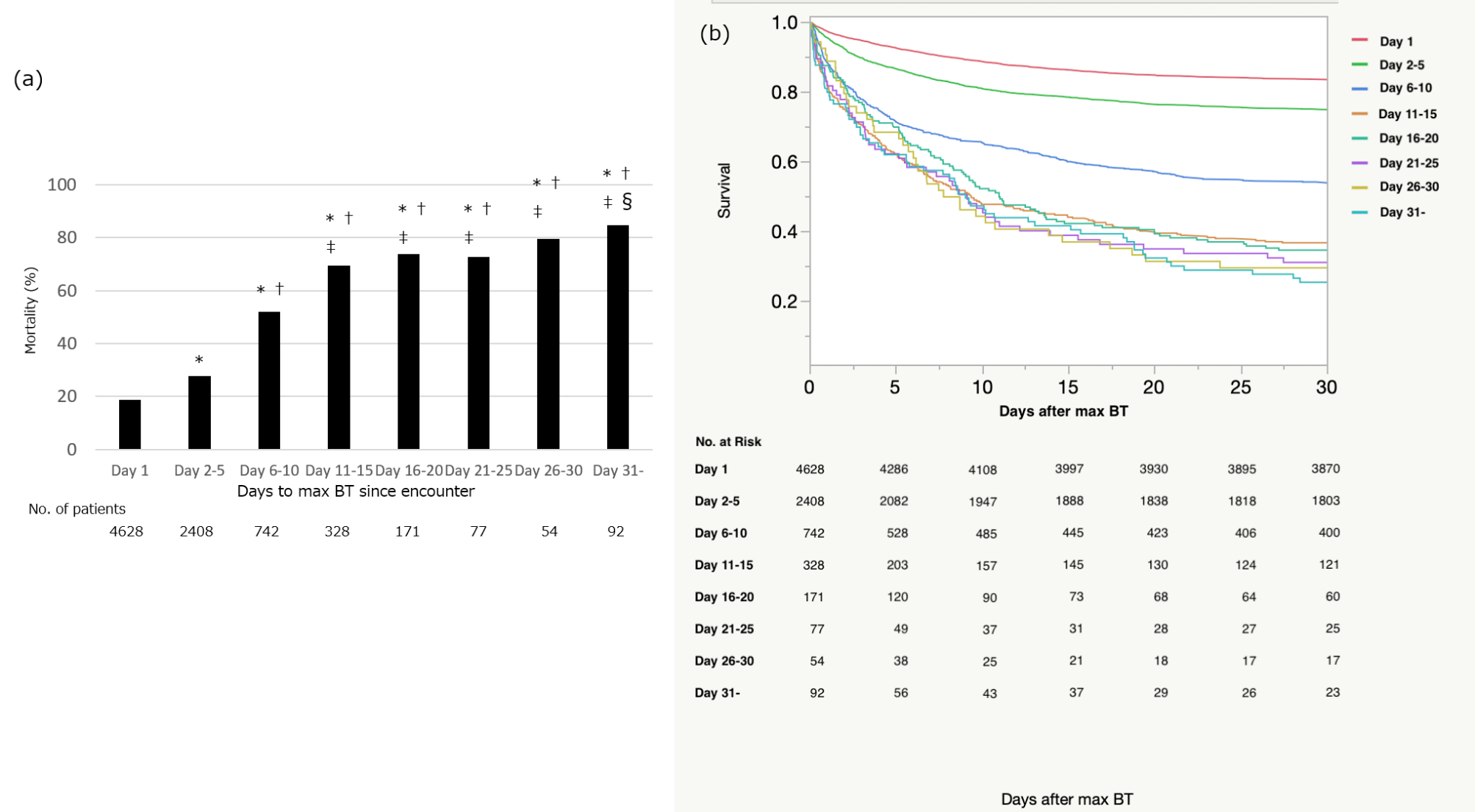


Patients were categorized based on the number of days until reaching maximum BT after initial encounter. Survival curves were drawn from the time of maximum BT with an assumption that discharged patients survived for 30 days (b). The p-value was <0.001 on Log-rank test.

* p<0.05 against day 1 group, † p<0.05 against day 2-5 group, ‡ p<0.05 against day　6-10 group, §p<0.05 against day 11-15 group on Chi-square with Holm’s test. BT = body temperature

**Supplement Figure 3: Maximum BT and mortality with and without vaccination in inpatients who were discharged home or expired.**


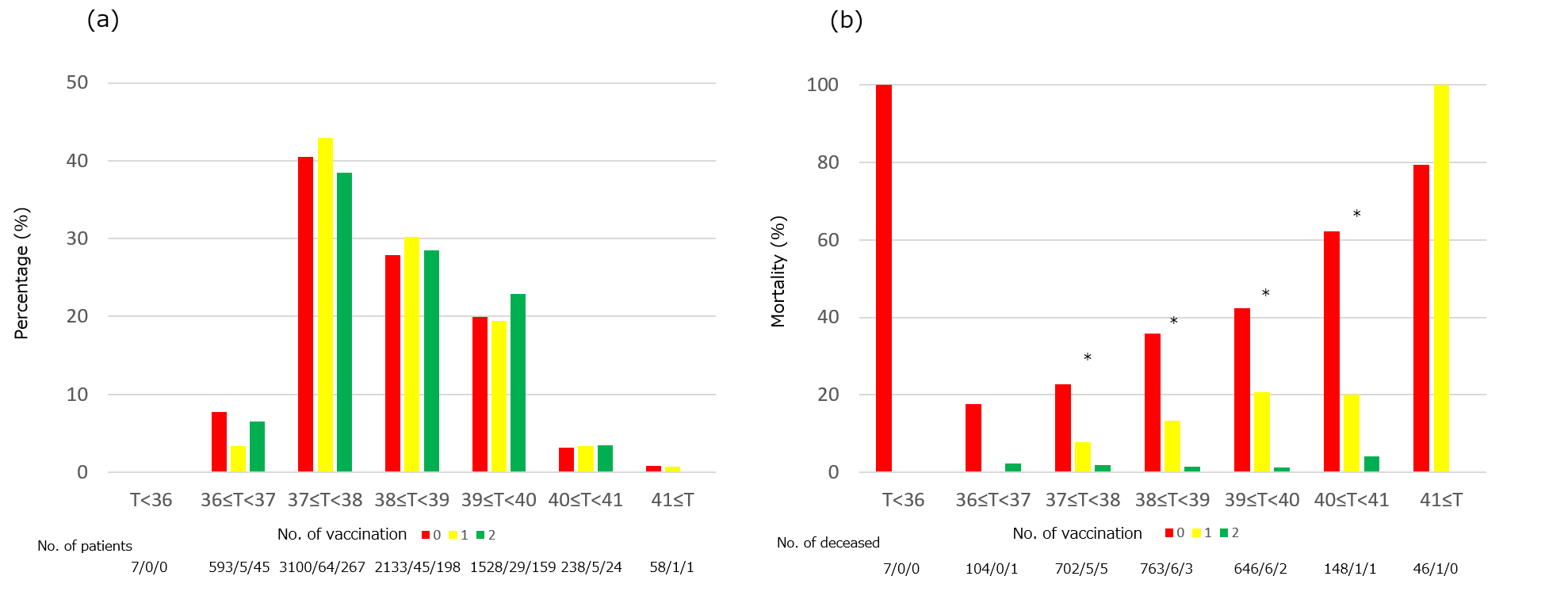


(a) Distribution of maximum BT and (b) mortality in patients grouped by vaccination and maximum BT. The distribution of maximum BT for each vaccination status did not show significant differences. In contrast, mortality was significantly reduced for each maximum BT category when the patients were vaccinated. Chi-square test for the entire group in (a) returned a p-value of 0.34. In (b), * demonstrates p<0.05 among the three vaccination groups. The p-value in 36 ≤maximum BT<37 °C was 0.17. The p-value in maximum BT ≥41 °C was 0.14.

BT = body temperature

**Supplemental table 1: Factors associated with maximum body temperature in all inpatients**

|  | Regression coefficient (95% CI) | p-value |
| --- | --- | --- |
| Age | -0.16 (-0.18 ~ -0.14) | < 0.0001 |
| Sex (male) | 0.12 (0.10 ~ 0.14) | < 0.0001 |
| Asthma | -0.0003 (-0.04 ~ 0.04) | 0.98 |
| Chronic obstructive pulmonary disease | -0.03 (-0.07 ~ 0.02) | 0.16 |
| Hypertension | 0.03 (0.01 ~ 0.48) | 0.0030 |
| Obstructive sleep apnea | 0.07 (0.01 ~ 0.13) | 0.017 |
| Diabetes | 0.03 (0.004 ~ 0.05) | 0.018 |
| Chronic kidney disease | 0.07 (0.04 ~ 0.10) | <0.0001 |
| Human immunodeficiency virus infection | 0.03 (-0.005 ~ 0.10) | 0.46 |
| Cancer | 0.09 (0.05 ~ 0.12) | <0.0001 |
| Coronary artery disease | -0.03 (-0.05 ~ -0.001) | 0.042 |
| Atrial fibrillation | -0.04 (-0.07 ~ -0.004) | 0.024 |
| Heart failure | -0.02 (-0.05 ~ 0.02) | 0.32 |
| Viral hepatitis | 0.05 (-0.05 ~ 0.14) | 0.31 |
| Alcoholic or nonalcoholic liver disease | 0.06 (-0.001 ~ 0.11) | 0.049 |
| Crohn's disease | -0.07 (-0.23 ~ 0.08) | 0.35 |
| Ulcerative colitis | 0.08 (-0.06 ~ 0.22) | 0.25 |
| Peripheral vascular disease | -0.003 (-0.05 ~ 0.04) | 0.89 |
| Body mass index | 0.25 (0.16 ~ 0.34) | <0.0001 |
| Vaccination 0→1 | 0.06 (-0.08 ~ 0.19) | 0.40 |
| Vaccination 1→2 | 0.009 (-0.14 ~ 0.16) | 0.90 |
| Linear regression analysis for maximum body temperature. CI = confidence interval | | |

**Supplemental table 2: Logistic regression analysis for mortality using factors associated with maximum BT in all inpatients**

|  | Odds ratio (95% CI) | p-value |
| --- | --- | --- |
| Maximum BT | 1.94 (1.83 ~ 2.05) | <0.0001 |
| Age | 1.07 (1.06 ~ 1.07) | <0.0001 |
| Sex (male) | 1.25 (1.12 – 1.39) | <0.0001 |
| Chronic kidney disease | 1.47 (1.26 ~ 1.71) | <0.0001 |
| Body mass index | 1.03 (1.02 ~ 1.03) | <0.0001 |
| Hypertension | 0.80 (0.71 ~ 0.90) | 0.0003 |
| Alcoholic or nonalcoholic liver disease | 1.63 (1.22 ~ 2.15) | 0.0008 |
| Coronary artery disease | 1.26 (1.09 ~ 1.45) | 0.0013 |
| Atrial fibrillation | 1.27 (1.08 ~ 1.50) | 0.0042 |
| Cancer | 1.09 (0.92 ~ 1.28) | 0.32 |
| Obstructive sleep apnea | 0.87 (0.62 ~ 1.21) | 0.42 |
| Diabetes | 1.05 (0.92 ~ 1.19) | 0.47 |
| Maximum BT and parameters with p<0.05 in Supplemental Table 1 were included in the analysis. BT = body temperature, CI = confidence interval | | |

**Supplemental Table 3: Patient characteristics in Dataset 3 including inpatients who were discharged home or expired**

| Patients number | 8,500 |
| --- | --- |
| Age (SD) y.o. | 62.96 (17.99) |
| Sex (male, %) | 4616 (54.3%) |
| Maximum temperature (Celsius, SD) | 38.20 (0.97) |
| Asthma | 520 (6.1%) |
| Chronic obstructive pulmonary disease | 365 (4.3%) |
| Hypertension | 2,976 (35.0%) |
| Obstructive sleep apnea | 209 (2.5%) |
| Diabetes mellitus | 1,937 (22.8%) |
| Chronic kidney disease | 950 (11.2%) |
| Human immunodeficiency virus infection | 147 (1.7%) |
| Cancer | 759 (8.9%) |
| Coronary artery disease | 1,192 (14.0%) |
| Atrial fibrillation | 676 (8.0%) |
| Heart failure | 747 (8.8%) |
| Viral hepatitis | 89 (1.0%) |
| Alcoholic or nonalcoholic liver disease | 251 (3.0%) |
| Crohn's disease | 33 (0.4%) |
| Ulcerative colitis | 41 (0.5%) |
| Peripheral vascular disease | 365 (4.3%) |
| Body mass index, (kg/m^2^, SD) | 28.97 (7.54) |
| Vaccination |  |
| 0 | 7,657 (90.1%) |
| 1 | 149 (1.8%) |
| 2 | 694 (8.2%) |

**Supplemental Table 4: Logistic regression analyses for inpatient death in inpatients who were discharged home or expired**

|  | **Univariate analysis** |  |  | **Multivariate analysis** |  |
| --- | --- | --- | --- | --- | --- |
|  | Odds ratio (95% CI) | p-value |  | Odds ratio (95% CI) | p-value |
| Maximum BT | 1.62 (1.54 ~ 1.71) | <0.0001 |  | 2.21 (2.07 ~ 2.37) | <0.0001 |
| Age | 1.06 (1.06 ~ 1.07) | <0.0001 |  | 1.08 (1.07 ~ 1.08) | <0.0001 |
| Sex (male) | 1.12 (1.02 ~ 1.23) | 0.02 |  | 1.17 (1.04 ~ 1.32) | 0.0009 |
| Asthma | 0.61 (0.49 ~ 0.76) | <0.0001 |  | 0.72 (0.54 ~ 0.94) | 0.02 |
| Chronic obstructive pulmonary disease | 1.95 (1.57 ~ 2.41) | <0.0001 |  | 1.37 (1.05 ~ 1.79) | 0.02 |
| Hypertension | 1.52 (1.38 ~ 1.67) | <0.0001 |  | 0.82 (0.71 ~ 0.95) | 0.006 |
| Obstructive sleep apnea | 0.93 (0.68 ~ 1.25) | 0.62 |  | - | - |
| Diabetes | 1.33 (1.20 ~ 1.49) | <0.0001 |  | 1.21 (1.04 ~ 1.40) | 0.01 |
| Chronic kidney disease | 2.03 (1.77 ~ 2.34) | <0.0001 |  | 1.93 (1.60 ~ 2.33) | <0.0001 |
| Human immunodeficiency virus infection | 0.71 (0.47 ~ 1.04) | 0.08 |  | - | - |
| Cancer | 1.43 (1.23 ~ 1.68) | <0.0001 |  | 1.36 (1.12 ~ 1.64) | 0.002 |
| Coronary artery disease | 1.96 (1.73 ~ 2.23) | <0.0001 |  | 1.28 (1.08 ~ 1.51) | 0.004 |
| Atrial fibrillation | 2.28 (1.95 ~ 2.68) | <0.0001 |  | 1.43 (1.17 ~1.76) | 0.0005 |
| Heart failure | 1.83 (1.57 ~ 2.13) | <0.0001 |  | 1.13 (0.91 ~ 1.39) | 0.27 |
| Viral hepatitis | 0.76 (0.46 ~ 1.22) | 0.27 |  | - | - |
| Alcoholic or nonalcoholic liver disease | 1.18 (0.90 ~ 1.54) | 0.22 |  | - | - |
| Crohn's disease | 0.08 (0.004 ~ 0.36) | <0.0001 |  | 0.15 (0.01 ~ 0.84) | 0.03 |
| Ulcerative colitis | 0.27 (0.08 ~ 0.66) | 0.003 |  | 0.51 (1.40 ~ 1.43) | 0.21 |
| Peripheral vascular disease | 1.52 (1.22 ~ 1.88) | 0.0002 |  | 0.84 (0.64 ~ 1.11) | 0.22 |
| Body mass index | 0.99 (0.98 ~ 0.99) | <0.0001 |  | 1.02 (1.01 ~ 1.03) | <0.0001 |
| Vaccination 0→1 | 0.32 (0.19 ~ 0.50) | <0.0001 |  | 0.21 (0.12 ~ 0.34) | <0.0001 |
| Vaccination 1→2 | 0.12 (0.056 ~ 0.25) | <0.0001 |  | 0.11 (0.05~ 0.25) | <0.0001 |
| Simple logistic regression (a) and multivariate logistic regression analysis (b) using the significant parameters in (a). BT = body temperature, CI = confidence interval | | | | | |
|  |  |  |  |  |  |
|  |  |  |  |  |  |

**Supplemental table 5: Factors associated with maximum body temperature in inpatients who were discharged home or expired**

|  | Regression coefficient (95% CI) | p-value |
| --- | --- | --- |
| Age | -0.08 (-0.12 ~ -0.03) | 0.003 |
| Sex (male) | 0.13 (0.11 ~ 0.15) | < 0.0001 |
| Asthma | 0.002 (-0.04 ~ 0.04) | 0.93 |
| Chronic obstructive pulmonary disease | -0.02 (-0.07 ~ 0.02) | 0.40 |
| Hypertension | 0.04 (0.02 ~ 0.06) | 0.0005 |
| Obstructive sleep apnea | 0.06 (-0.01 ~ 0.12) | 0.10 |
| Diabetes | 0.02 (-0.005 ~ 0.04) | 0.11 |
| Chronic kidney disease | 0.08 (0.05 ~ 0.11) | <0.0001 |
| Human immunodeficiency virus infection | 0.04 (-0.04 ~ 0.11) | 0.38 |
| Cancer | 0.10 (0.06 ~ 0.13) | <0.0001 |
| Coronary artery disease | -0.02 (-0.05 ~ -0.01) | 0.29 |
| Atrial fibrillation | -0.04 (-0.08 ~ -0.003) | 0.04 |
| Heart failure | -0.01 (-0.05 ~ 0.02) | 0.45 |
| Viral hepatitis | 0.04 (-0.06 ~ 0.14) | 0.44 |
| Alcoholic or nonalcoholic liver disease | 0.06 (-0.006 ~ 0.12) | 0.08 |
| Crohn's disease | -0.05 (-0.21 ~ 0.12) | 0.58 |
| Ulcerative colitis | 0.07 (-0.08 ~ 0.22) | 0.33 |
| Peripheral vascular disease | -0.002 (-0.05 ~ 0.05) | 0.94 |
| Body mass index | 0.20 (0.10 ~ 0.29) | <0.0001 |
| Vaccination 0→1 | 0.05 (-0.11 ~ 0.21) | 0.55 |
| Vaccination 1→2 | 0.01 (-0.16 ~ 0.18) | 0.89 |

**Supplemental table 6: Logistic regression analysis for mortality using factors associated with maximum BT in inpatients who were discharged home or expired**

|  | Odd ratio (95% CI) | p-value |
| --- | --- | --- |
| Maximum BT | 2.13 (2.00 ~ 2.27) | <0.0001 |
| Age | 1.08 (1.08 ~ 1.09) | <0.0001 |
| Sex (male) | 1.23 (1.09 – 1.38) | <0.0001 |
| Chronic kidney disease | 1.73 (1.46 ~ 2.05) | <0.0001 |
| Body mass index | 1.02 (1.01 ~ 1.03) | <0.0001 |
| Atrial fibrillation | 1.41 (1.17 ~ 1.69) | 0.0002 |
| Hypertension | 0.84 (0.75 ~ 0.95) | 0.007 |
| Cancer | 1.08 (0.90 ~ 1.28) | 0.42 |
| Maximum BT and parameters with p<0.05 in Supplemental Table 1 were included in the analysis. BT = body temperature, CI = confidence interval | | |

**Supplemental table 7: Chi-square test p value without correction for analysis for initial body temperature and mortality (Figure 2a)**

|  | **<36** | **36-37** | **37-38** | **38-39** | **39-40** | **>40** |
| --- | --- | --- | --- | --- | --- | --- |
| **<36** |  | <0.0001 | <0.0001 | <0.0001 | 0.0011 | 0.8096 |
| **36-37** |  |  | 0.2069 | <0.0001 | <0.0001 | 0.022 |
| **37-38** |  |  |  | <0.0001 | <0.0001 | 0.0329 |
| **38-39** |  |  |  |  | 0.2607 | 0.2817 |
| **39-40** |  |  |  |  |  | 0.442 |
| **>40** |  |  |  |  |  |  |

**Supplemental table 8: Pairwise Chi-square p value and Holm's corrected alpha of analysis for initial body temperature and mortality (Figure 2a)**

| **Original alpha = 0.05** |  |  | |  |  |
| --- | --- | --- | --- | --- | --- |
| **Pair** | **p value** | **Holm's corrected alpha** | | **m-i+1** | **Accept/Reject null hypothesis (NH)** |
| <36 vs 36-37 | <0.0001 | 0.00333 | | 15 | Reject NH |
| <36 vs 37-38 | <0.0001 | 0.00357 | | 14 | Reject NH |
| <36 vs 38-39 | <0.0001 | 0.00385 | | 13 | Reject NH |
| 36-37 vs 38-39 | <0.0001 | 0.00417 | | 12 | Reject NH |
| 36-37 vs 39-40 | <0.0001 | 0.00455 | | 11 | Reject NH |
| 37-38 vs 38-39 | <0.0001 | 0.005 | | 10 | Reject NH |
| 37-38 vs 39-40 | <0.0001 | 0.00556 | | 9 | Reject NH |
| <36 vs 39-40 | 0.0011 | 0.00625 | | 8 | Reject NH |
| 36-37 vs >40 | 0.022 | 0.00714 | | 7 | Accept NH |
| 37-37 vs >40 | 0.0329 | 0.00833 | | 6 | Accept NH |
| 36-37 vs 37-38 | 0.2069 | 0.01 | | 5 | Accept NH |
| 38-39 vs 39-40 | 0.2607 | 0.0125 | | 4 | Accept NH |
| 38-39 vs >40 | 0.2817 | 0.0167 | | 3 | Accept NH |
| 39-40 vs >40 | 0.442 | 0.025 | | 2 | Accept NH |
| <36 vs >40 | 0.8096 | 0.05 | | 1 | Accept NH |
| Each entry on the table corresponds to a null hypothesis of the form "There are no statistically significant differences between two temperature groups. | | | | | |
| Hypotheses are in ascending order of respective p value. | | | |  |  |
| Corrected alpha is calculated as: | |  | |  |  |
| corrected_alpha = alpha/(m-i+1) | |  | |  |  |
| where |  |  | |  |  |
| alpha = 0.05 (initial significance threshold), | | | |  |  |
| m = 15 (number of hypotheses) and 1 ≤ i ≤ 15 (position of hypotheses) | | |  |  |  |

**Supplemental table 9: Chi-square test p value without correction for analysis for maximum body temperature and mortality (Figure 2b)**

|  | **<36** | **36-37** | **37-38** | **38-39** | **39-40** | **40-41** | **>41** |
| --- | --- | --- | --- | --- | --- | --- | --- |
| **<36** |  | <.0001 | <.0001 | <.0001 | <.0001 | 0.0046 | 0.0464 |
| **36-37** |  |  | 0.0998 | <.0001 | <.0001 | <.0001 | <.0001 |
| **37-38** |  |  |  | <.0001 | <.0001 | <.0001 | <.0001 |
| **38-39** |  |  |  |  | <.0001 | <.0001 | <.0001 |
| **39-40** |  |  |  |  |  | <.0001 | <.0001 |
| **40-41** |  |  |  |  |  |  | 0.0093 |
| **>41** |  |  |  |  |  |  |  |

**Supplemental table 10: Pairwise Chi-square p value and Holm's corrected alpha of analysis for maximum body temperature and mortality (Figure 2b)**

| **Original alpha = 0.05** | |  |  |  |
| --- | --- | --- | --- | --- |
| **Pair** | **p value** | **Holm's corrected alpha** | **m-i+1** | **Accept/Reject null hypothesis (NH)** |
| <36 vs 36-37 | <0.0001 | 0.00238 | 21 | Reject NH |
| <36 vs 37-38 | <0.0001 | 0.0025 | 20 | Reject NH |
| <36 vs 38-39 | <0.0001 | 0.00263 | 19 | Reject NH |
| <36 vs 39-40 | <0.0001 | 0.00278 | 18 | Reject NH |
| 36-37 vs 38-39 | <0.0001 | 0.00294 | 17 | Reject NH |
| 36-37 vs 39-40 | <0.0001 | 0.00313 | 16 | Reject NH |
| 36-37 vs 40-41 | <0.0001 | 0.00333 | 15 | Reject NH |
| 36-37 vs >41 | <0.0001 | 0.00357 | 14 | Reject NH |
| 37-38 vs 38-39 | <0.0001 | 0.00385 | 13 | Reject NH |
| 37-38 vs 39-40 | <0.0001 | 0.00417 | 12 | Reject NH |
| 37-38 vs 40-41 | <0.0001 | 0.00455 | 11 | Reject NH |
| 37-38 vs >41 | <0.0001 | 0.005 | 10 | Reject NH |
| 38-39 vs 39-40 | <0.0001 | 0.00556 | 9 | Reject NH |
| 38-39 vs 40-41 | <0.0001 | 0.00625 | 8 | Reject NH |
| 38-39 vs >41 | <0.0001 | 0.00714 | 7 | Reject NH |
| 39-40 vs 40-41 | <0.0001 | 0.00833 | 6 | Reject NH |
| 39-40 vs >41 | <0.0001 | 0.01 | 5 | Reject NH |
| <36 vs 40-41 | 0.0046 | 0.0125 | 4 | Reject NH |
| 40-41 vs >41 | 0.0093 | 0.0167 | 3 | Reject NH |
| <36 vs >41 | 0.0464 | 0.025 | 2 | Accept NH |
| 36-37 vs 37-38 | 0.0998 | 0.05 | 1 | Accept NH |
| Each entry on the table corresponds to a null hypothesis of the form "There are no statistically significant differences between two temperature groups. | | | | |
| Hypotheses are in ascending order of respective p value. | | |  |  |
| Corrected alpha is calculated as: | |  |  |  |
| corrected_alpha = alpha/(m-i+1) | |  |  |  |
| where |  |  |  |  |
| alpha = 0.05 (initial significance threshold), | | |  |  |
| m = 21 (number of hypotheses) and 1 ≤ i ≤ 21 (position of hypotheses) | | |  |  |

**Supplemental table 11: Chi-square test p value without correction for analysis for analysis for hospital days of maximum body temperature and mortality (Figure 3)**

|  | **1** | **2-5** | **6-10** | **11-15** | **16-20** | **21-25** | **26-30** | **>31** |
| --- | --- | --- | --- | --- | --- | --- | --- | --- |
| **1** |  | <.0001 | <.0001 | <.0001 | <.0001 | <.0001 | <.0001 | <.0001 |
| **2-5** |  |  | <.0001 | <.0001 | <.0001 | <.0001 | <.0001 | <.0001 |
| **6-10** |  |  |  | <.0001 | <.0001 | 0.106 | 0.0173 | 0.0005 |
| **11-15** |  |  |  |  | 0.8685 | 0.2008 | 0.8845 | 0.8326 |
| **16-20** |  |  |  |  |  | 0.1949 | 0.8093 | 0.948 |
| **21-25** |  |  |  |  |  |  | 0.4233 | 0.2174 |
| **26-30** |  |  |  |  |  |  |  | 0.7842 |
| **>31** |  |  |  |  |  |  |  |  |

**Supplemental table 12: Pairwise Chi-square p value and Holm's corrected alpha of analysis for hospital days of maximum body temperature and mortality (Figure 3)**

| **Original alpha = 0.05** | |  |  |  |
| --- | --- | --- | --- | --- |
| **Pair** | **p value** | **Holm's corrected alpha** | **m-i+1** | **Accept/Reject null hypothesis (NH)** |
| 1 vs 2-5 | <0.0001 | 0.00179 | 28 | Reject NH |
| 1 vs 6-10 | <0.0001 | 0.00185 | 27 | Reject NH |
| 1 vs 11-15 | <0.0001 | 0.00192 | 26 | Reject NH |
| 1 vs 16-20 | <0.0001 | 0.002 | 25 | Reject NH |
| 1 vs 21-25 | <0.0001 | 0.00208 | 24 | Reject NH |
| 1 vs 26-30 | <0.0001 | 0.00217 | 23 | Reject NH |
| 1 vs >31 | <0.0001 | 0.00227 | 22 | Reject NH |
| 2-5 vs 6-10 | <0.0001 | 0.00238 | 21 | Reject NH |
| 2-5 vs 11-15 | <0.0001 | 0.0025 | 20 | Reject NH |
| 2-5 vs 16-20 | <0.0001 | 0.00263 | 19 | Reject NH |
| 2-5 vs 21-25 | <0.0001 | 0.00278 | 18 | Reject NH |
| 2-5 vs 26-30 | <0.0001 | 0.00294 | 17 | Reject NH |
| 2-5 vs >31 | <0.0001 | 0.00313 | 16 | Reject NH |
| 6-10 vs 11-15 | <0.0001 | 0.00333 | 15 | Reject NH |
| 6-10 vs 16-20 | <0.0001 | 0.00357 | 14 | Reject NH |
| 6-10 vs >31 | 0.0005 | 0.00385 | 13 | Reject NH |
| 6-10 vs 26-30 | 0.0173 | 0.00417 | 12 | Accept NH |
| 6-10 vs 21-25 | 0.106 | 0.00455 | 11 | Accept NH |
| 16-20 vs 21-25 | 0.1949 | 0.005 | 10 | Accept NH |
| 11-15 vs 21-25 | 0.2008 | 0.00556 | 9 | Accept NH |
| 21-25 vs >31 | 0.2174 | 0.00625 | 8 | Accept NH |
| 21-25 vs 26-30 | 0.4233 | 0.00714 | 7 | Accept NH |
| 26-30 vs >31 | 0.7842 | 0.00833 | 6 | Accept NH |
| 16-20 vs 26-30 | 0.8093 | 0.01 | 5 | Accept NH |
| 11-15 vs >31 | 0.8326 | 0.0125 | 4 | Accept NH |
| 11-15 vs 16-20 | 0.8685 | 0.0167 | 3 | Accept NH |
| 11-15 vs 26-30 | 0.8845 | 0.025 | 2 | Accept NH |
| 16-20 vs >31 | 0.948 | 0.05 | 1 | Accept NH |
| Each entry on the table corresponds to a null hypothesis of the form "There are no statistically significant differences between two temperature groups. | | | | |
| Hypotheses are in ascending order of respective p value. | | | |  |
| Corrected alpha is calculated as: | | |  |  |
| corrected_alpha = alpha/(m-i+1) | | |  |  |
| where |  |  |  |  |
| alpha = 0.05 (initial significance threshold), | | |  |  |
| m = 28 (number of hypotheses) and 1 ≤ i ≤ 28 (position of hypotheses) | | |  |  |

**Supplemental table 13: Pairwise Chi-square p value and Holm's corrected alpha of analysis for vaccination status and mortality (Figure 4b)**

| **Original alpha = 0.05** | | |  | |  |  |
| --- | --- | --- | --- | --- | --- | --- |
| **Pair** | | **p value** | **Holm's corrected alpha** | | **m-i+1** | **Accept/Reject null hypothesis (NH)** |
| 37-38 | | <0.0001 | 0.00833 | | 6 | Reject NH |
| 38-39 | | <0.0001 | 0.01 | | 5 | Reject NH |
| 39-40 | | <0.0001 | 0.0125 | | 4 | Reject NH |
| 40-41 | | <0.0001 | 0.0167 | | 3 | Reject NH |
| 36-37 | | 0.0223 | 0.025 | | 2 | Reject NH |
| >41 | | 0.0559 | 0.05 | | 1 | Accept NH |
| In <36 group, no patients took vaccination. | | |  | |  |  |
| Each entry on the table corresponds to a null hypothesis of the form "There are no statistically significant differences between two temperature groups. | | | | | | |
| Hypotheses are in ascending order of respective p value. | | | | | |  |
| Corrected alpha is calculated as: | | | | |  |  |
| corrected alpha = alpha/(m-i+1) | | | | |  |  |
| where | |  |  | |  |  |
| alpha = 0.05 (initial significance threshold), | | | | |  |  |
| m = 6 (number of hypotheses) and 1 ≤ i ≤ 6 (position of hypotheses) | | | | |  |  |
|  |  | | |  |  |  |

**Supplemental table 14: Chi-square test p value without correction for analysis for maximum body temperature and mortality of inpatients who were discharged home or expired (Supplemental Figure 1)**

|  | **<36** | **36-37** | **37-38** | **38-39** | **39-40** | **40-41** | **>41** |
| --- | --- | --- | --- | --- | --- | --- | --- |
| **<36** |  | <.0001 | <.0001 | 0.0001 | 0.0008 | 0.0207 | 0.1701 |
| **36-37** |  |  | 0.0102 | <.0001 | <.0001 | <.0001 | <.0001 |
| **37-38** |  |  |  | <.0001 | <.0001 | <.0001 | <.0001 |
| **38-39** |  |  |  |  | 0.0002 | <.0001 | <.0001 |
| **39-40** |  |  |  |  |  | <.0001 | <.0001 |
| **40-41** |  |  |  |  |  |  | 0.0015 |
| **>41** |  |  |  |  |  |  |  |

**Supplemental table 15: Pairwise Chi-square p value and Holm's corrected alpha of analysis for maximum body temperature and mortality of inpatients who were discharged home or expired (Supplemental Figure 1)**

| **Original alpha = 0.05** | |  |  |  |
| --- | --- | --- | --- | --- |
| **Pair** | **p value** | **Holm's corrected alpha** | **m-i+1** | **Accept/Reject null hypothesis (NH)** |
| <36 vs 36-37 | <0.0001 | 0.00238 | 21 | Reject NH |
| <36 vs 37-38 | <0.0001 | 0.0025 | 20 | Reject NH |
| 36-37 vs 38-39 | <0.0001 | 0.00263 | 19 | Reject NH |
| 36-37 vs 39-40 | <0.0001 | 0.00278 | 18 | Reject NH |
| 36-37 vs 40-41 | <0.0001 | 0.00294 | 17 | Reject NH |
| 36-37 vs >41 | <0.0001 | 0.00313 | 16 | Reject NH |
| 37-38 vs 38-39 | <0.0001 | 0.00333 | 15 | Reject NH |
| 37-38 vs 39-40 | <0.0001 | 0.00357 | 14 | Reject NH |
| 37-38 vs 40-41 | <0.0001 | 0.00385 | 13 | Reject NH |
| 37-38 vs >41 | <0.0001 | 0.00417 | 12 | Reject NH |
| 38-39 vs 40-41 | <0.0001 | 0.00455 | 11 | Reject NH |
| 38-39 vs >41 | <0.0001 | 0.005 | 10 | Reject NH |
| 39-40 vs 40-41 | <0.0001 | 0.00556 | 9 | Reject NH |
| 39-40 vs >41 | <0.0001 | 0.00625 | 8 | Reject NH |
| <36 vs 38-39 | 0.0001 | 0.00714 | 7 | Reject NH |
| 38-39 vs 39-40 | 0.0002 | 0.00833 | 6 | Reject NH |
| <36 vs 39-40 | 0.0008 | 0.01 | 5 | Reject NH |
| 40-41 vs >41 | 0.0015 | 0.0125 | 4 | Reject NH |
| 36-37 vs 37-38 | 0.0102 | 0.0167 | 3 | Reject NH |
| <36 vs 40-41 | 0.0207 | 0.025 | 2 | Reject NH |
| <36 vs >41 | 0.1701 | 0.05 | 1 | Accept NH |
| Each entry on the table corresponds to a null hypothesis of the form "There are no statistically significant differences between two temperature groups. | | | | |
| Hypotheses are in ascending order of respective p value. | | |  |  |
| Corrected alpha is calculated as: | |  |  |  |
| corrected_alpha = alpha/(m-i+1) | |  |  |  |
| where |  |  |  |  |
| alpha = 0.05 (initial significance threshold), | | |  |  |
| m = 21 (number of hypotheses) and 1 ≤ i ≤ 21 (position of hypotheses) | | |  |  |

**Supplemental table 16: Chi-square test p value without correction for** **analysis for hospital days of maximum body temperature and mortality of inpatients who were discharged home or expired (Supplemental Figure 2)**

|  | **1** | **2-5** | **6-10** | **11-15** | **16-20** | **21-25** | **26-30** | **>31** |
| --- | --- | --- | --- | --- | --- | --- | --- | --- |
| **1** |  | <.0001 | <.0001 | <.0001 | <.0001 | <.0001 | <.0001 | <.0001 |
| **2-5** |  |  | <.0001 | <.0001 | <.0001 | <.0001 | <.0001 | <.0001 |
| **6-10** |  |  |  | <.0001 | <.0001 | 0.0005 | <.0001 | <.0001 |
| **11-15** |  |  |  |  | 0.33 | 0.5791 | 0.1292 | 0.0036 |
| **16-20** |  |  |  |  |  | 0.8746 | 0.3784 | 0.0396 |
| **21-25** |  |  |  |  |  |  | 0.3654 | 0.0541 |
| **26-30** |  |  |  |  |  |  |  | 0.4249 |
| **>31** |  |  |  |  |  |  |  |  |

**Supplemental table 17: Pairwise Chi-square p value and Holm's corrected alpha of analysis for hospital days of maximum body temperature and mortality of inpatients who were discharged home or expired (Supplemental Figure 2)**

| **Original alpha = 0.05** | |  |  |  |
| --- | --- | --- | --- | --- |
| **Pair** | **p value** | **Holm's corrected alpha** | **m-i+1** | **Accept/Reject null hypothesis (NH)** |
| 1 vs 2-5 | <0.0001 | 0.00179 | 28 | Reject NH |
| 1 vs 6-10 | <0.0001 | 0.00185 | 27 | Reject NH |
| 1 vs 11-15 | <0.0001 | 0.00192 | 26 | Reject NH |
| 1 vs 16-20 | <0.0001 | 0.002 | 25 | Reject NH |
| 1 vs 21-25 | <0.0001 | 0.00208 | 24 | Reject NH |
| 1 vs 26-30 | <0.0001 | 0.00217 | 23 | Reject NH |
| 1 vs >31 | <0.0001 | 0.00227 | 22 | Reject NH |
| 2-5 vs 6-10 | <0.0001 | 0.00238 | 21 | Reject NH |
| 2-5 vs 11-15 | <0.0001 | 0.0025 | 20 | Reject NH |
| 2-5 vs 16-20 | <0.0001 | 0.00263 | 19 | Reject NH |
| 2-5 vs 21-25 | <0.0001 | 0.00278 | 18 | Reject NH |
| 2-5 vs 26-30 | <0.0001 | 0.00294 | 17 | Reject NH |
| 2-5 vs >31 | <0.0001 | 0.00313 | 16 | Reject NH |
| 6-10 vs 11-15 | <0.0001 | 0.00333 | 15 | Reject NH |
| 6-10 vs 16-20 | <0.0001 | 0.00357 | 14 | Reject NH |
| 6-10 vs 26-30 | <0.0001 | 0.00385 | 13 | Reject NH |
| 6-10 vs >31 | <0.0001 | 0.00417 | 12 | Reject NH |
| 6-10 vs 21-25 | 0.0005 | 0.00455 | 11 | Reject NH |
| 16-20 vs 21-25 | 0.0036 | 0.005 | 10 | Reject NH |
| 11-15 vs 21-25 | 0.0396 | 0.00556 | 9 | Accept NH |
| 21-25 vs >31 | 0.0541 | 0.00625 | 8 | Accept NH |
| 21-25 vs 26-30 | 0.1292 | 0.00714 | 7 | Accept NH |
| 26-30 vs >31 | 0.33 | 0.00833 | 6 | Accept NH |
| 16-20 vs 26-30 | 0.3654 | 0.01 | 5 | Accept NH |
| 11-15 vs >31 | 0.3784 | 0.0125 | 4 | Accept NH |
| 11-15 vs 16-20 | 0.4249 | 0.0167 | 3 | Accept NH |
| 11-15 vs 26-30 | 0.5791 | 0.025 | 2 | Accept NH |
| 16-20 vs >31 | 0.8746 | 0.05 | 1 | Accept NH |
| Each entry on the table corresponds to a null hypothesis of the form "There are no statistically significant differences between two temperature groups. | | | | |
| Hypotheses are in ascending order of respective p value. | | | |  |
| Corrected alpha is calculated as: | | |  |  |
| corrected_alpha = alpha/(m-i+1) | | |  |  |
| where |  |  |  |  |
| alpha = 0.05 (initial significance threshold), | | |  |  |
| m = 28 (number of hypotheses) and 1 ≤ i ≤ 28 (position of hypotheses) | | |  |  |

**Supplemental table 18: Pairwise Chi-square p value and Holm's corrected alpha of analysis for vaccination status and mortality of inpatients who were discharged home or expired.**

| **Original alpha = 0.05** | |  |  |  |
| --- | --- | --- | --- | --- |
| **Pair** | **p value** | **Holm's corrected alpha** | **m-i+1** | **Accept/Reject null hypothesis (NH)** |
| 37-38 | <0.0001 | 0.00833 | 6 | Reject NH |
| 38-39 | <0.0001 | 0.01 | 5 | Reject NH |
| 39-40 | <0.0001 | 0.0125 | 4 | Reject NH |
| 40-41 | <0.0001 | 0.0167 | 3 | Reject NH |
| >41 | 0.1405 | 0.025 | 2 | Accept NH |
| 36-37 | 0.169 | 0.05 | 1 | Accept NH |
| In <36 group, no patients took vaccination. | |  |  |  |
| Each entry on the table corresponds to a null hypothesis of the form "There are no statistically significant differences between two temperature groups. | | | | |
| Hypotheses are in ascending order of respective p value. | | | |  |
| Corrected alpha is calculated as: | | |  |  |
| corrected_alpha = alpha/(m-i+1) | | |  |  |
| where |  |  |  |  |
| alpha = 0.05 (initial significance threshold), | | |  |  |
| m = 6 (number of hypotheses) and 1 ≤ i ≤ 6 (position of hypotheses) | | |  |  |
